# People Seeking Treatment for Meth/Amphetamine Use Are Getting Older and Treatment Services Need to Adapt

**DOI:** 10.1111/dar.70238

**Published:** 2026-08-18

**Authors:** Liam S. Acheson, Krista J. Siefried, Brendan Clifford, Maureen Steele, Kathryn Fletcher, Nadine Ezard

**Affiliations:** ^1^ National Centre for Clinical Research on Emerging Drugs UNSW Sydney Sydney Australia; ^2^ Alcohol and Drug Service St Vincent's Hospital Sydney Sydney Australia; ^3^ National Drug and Alcohol Research Centre UNSW Sydney Sydney Australia; ^4^ New South Wales Drug and Alcohol Clinical Research and Improvement Network New South Wales Ministry of Health Sydney Australia; ^5^ Centre for Alcohol and Other Drugs New South Wales Ministry of Health Sydney Australia

**Keywords:** age, ageing, amphetamine, methamphetamine, treatment

## Abstract

**Introduction:**

Harms associated with meth/amphetamine use increase with cumulative exposure and overlap with age‐related health conditions. Understanding demographic shifts in drug treatment populations is required to ensure services meet evolving needs.

**Methods:**

We analysed data from the Australian Institute of Health and Welfare's Alcohol and Other Drug Treatment Services National Minimum Data Set, including all treatment episodes where meth/amphetamine was the primary drug of concern from 2003–04 (first data available) to 2023–24. Weighted linear regression assessed changes in age distribution over time.

**Results:**

The analysis included 697,280 treatment episodes, 35% (*n* = 245,782) of whom were female. In 2003–04, people aged 20–29 years were the largest group accessing treatment (*n* = 6835, 48%), but by 2023–24 those aged 30–39 were the largest group seeking treatment for meth/amphetamine use (*n* = 258,063, 39%, *p* < 0.001). Over the last 20 years, the most pronounced increases occurred among adults over the age of 50 years, with treatment episodes increasing 16‐fold (from 64 to 4313, *p* < 0.001) among those aged 50–59 and 13‐fold (from 11 to 609, *p* < 0.001) among those aged 60 years and over.

**Discussion and Conclusions:**

The population seeking treatment for meth/amphetamine use disorder is ageing, outpacing the general Australian population. The absence of policies and practice guidance for management of meth/amphetamine use disorder and coexisting conditions among older people represents a critical gap. Treatment services must adapt to provide age‐appropriate care for older Australians, as the proportion of people over the age of 50 accessing treatment continues to grow.

## Key Point Summary

- Meth/amphetamine associated harms increase over time, and harms overlap with age‐related comorbidities.
- The population seeking treatment for meth/amphetamine use in Australia is ageing, and is likely to continue to do so.
- Treatment guidelines do not address concurrent age and meth/amphetamine‐related conditions, and treatment services need to adapt to a changing population.

## 1 Introduction

Amphetamines, predominantly in the form of methamphetamine, are the most widely consumed synthetic stimulant in the world, used by an estimated 30 million people [1], and are now the most common illicit drug for which treatment is sought in Australia [2]. While data sets refer to ‘amphetamine’ in Australia, the vast majority of amphetamines available are methamphetamine [3], and both terms generally refer to methamphetamine in the Australian context. For the purpose of this paper, we will use the term meth/amphetamine to reflect this. The harms associated with meth/amphetamine use have increased over the past 10 years [4], reflected by an increasing demand for treatment [1]. Regular meth/amphetamine use may lead to meth/amphetamine use disorder (MAUD). Regular meth/amphetamine use and MAUD are associated with depression, anxiety, psychosis, cardiovascular disease (including ischaemic heart disease, cardiomyopathy, pulmonary arterial hypertension), stroke and increased risk of sexually transmitted or blood‐borne viral infections [5].

The harms associated with meth/amphetamine use increase over time due to cumulative long‐term exposure [6]. While meth/amphetamine‐related morbidity and mortality are linked to frequency, intensity and use patterns, the risk of adverse outcomes increases over time due to compounding effects [7, 8]. There is significant overlap between meth/amphetamine harms and age‐related conditions, most notably cardiovascular and cerebrovascular comorbidities [9]. Cardiovascular disease in older people is common in, and may be a compounding factor of, meth/amphetamine overdose deaths [10], however limited information exists regarding meth/amphetamine use in older people in Australia. In 2021 it was noted that an increasing number of people aged 50+ have been accessing treatment for substance use in Australia, and this trend was also observed in people who use meth/amphetamine [11].

Australia has an ageing population. A total of 1 in 6 Australians is now over the age of 65 and this is expected to increase to 1 in 5 by 2060 [12]. Treatment services must adapt to the changing needs of the Australian population, and a clearer understanding of the demographics of those seeking treatment for meth/amphetamine use is required. This paper aims to use publicly available treatment data to characterise how the age of individuals seeking treatment for MAUD in Australia changes and examine trends over time.

## 2 Methods

This study reports on a secondary analysis of the Alcohol and Other Drug Treatment Services National Minimum Data Set (AODTS NMDS). AODTS NMDS represents the minimum data that public drug treatment services in Australia must collect and report on for all people attending treatment for alcohol and other drug use. It consists of over 40 data elements and forms a publicly available dataset managed by the Australian Institute of Health and Welfare.

Data were extracted from the publicly available Australian Institute of Health and Welfare AODTS NMDS data including demographic and treatment utilisation data for individuals reporting meth/amphetamine as their primary drug of concern in Australia from 2003–04 (first data available) to 2023–24. Sex was recorded as binary (male/female) and age was available only in aggregated groups (10–19, 20–29, 30–39, 40–49, 50–59 and 60+ years), which were used in the analysis.

### 2.1 Analysis

Data analysis was conducted in R (R Core Team, version 4.4.1, 2024). For each financial year from 2003–04 to 2023–24, we extracted the number of treatment episodes for meth/amphetamine as the principal drug of concern by age group (10–19, 20–29, 30–39, 40–49, 50–59 and 60+ years) and the total number of episodes. For each year and age group, we derived the proportion of episodes by dividing the age‐group count by the total number of episodes. To assess temporal trends in age distribution, we fitted separate linear regression models for each age group, with the proportion of episodes as the dependent variable and time (coded as years since 2003–04) as the independent variable. Weighted least squares was used, with weights equal to the total number of episodes in each year, to account for variation in annual sample size [13]. The regression slope coefficient represents the average annual change in the proportion (percentage points per year). Estimated slopes are reported with 95% confidence intervals and *p*‐values. Model assumptions were assessed using residual plots.

## 3 Results

We included data from 697,280 individual treatment episodes in Australian drug treatment services between 2003–04 and 2023–24. One‐third of treatment episodes were for people who were recorded as female (*n* = 245,782, 35%). The total number of treatment episodes increased from 14,208 in 2003–04 to 57,791 in 2023–24, and the raw number of treatment episodes increased for each age group over the 20‐year period.

**FIGURE 1:**
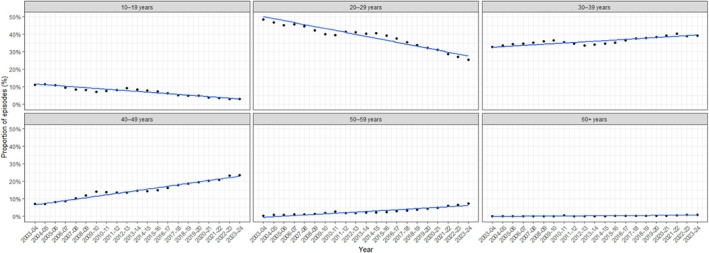
Proportion of treatment episodes with meth/amphetamine as primary drug of concern in Australia by individual age group, with line of best fit.

Weighted linear regression of age‐specific proportions on year showed well‐fitting linear trends across age groups (*R* ^2^ range 0.68–0.97). The proportion of episodes declined among younger age groups (10–19: −0.43 and 20–29: −1.12 percentage points per year), while increasing among older groups (30–39: +0.35, 40–49: +0.82, 50–59: +0.34, and 60+: +0.04 percentage points per year; all *p* < 0.001). This is equivalent to a 16‐fold increase in treatment episodes for people aged 50–59 and a 13‐fold increase for those 60+. Data are summarised in Table 1, and individual age group proportions with trend lines are presented in Figure 1.

**TABLE 1:** Summary of change in proportion of treatment episodes with meth/amphetamine as primary drug of concern in Australia by age group, 2003–04 to 2023–24.

Age group	Slope	95% CI	*p*	*R* ^2^
10–19	−0.0043	−0.0049, −0.0037	< 0.001	0.92
20–29	−0.0112	−0.0128, −0.0097	< 0.001	0.92
30–39	0.0035	0.0026, 0.0044	< 0.001	0.77
40–49	0.0082	0.0075, 0.0089	< 0.001	0.97
50–59	0.0034	0.0027, 0.0040	< 0.001	0.86
60+	0.0004	0.0003, 0.0006	< 0.001	0.68

Abbreviation: CI, confidence interval.

## 4 Discussion

Using 20 years of national treatment data, we identified a progressive shift over time in meth/amphetamine‐related treatment episodes from younger to older adults in Australia, with a significant decline among younger Australians and a corresponding increase among older age groups.

This study is limited as it only includes data from publicly funded drug and alcohol specialist treatment services and does not include services delivered by private providers. This data source also excludes services delivered by inpatient providers without specialist drug and alcohol services, such as acute care or psychiatric hospitals. These data report on treatment episode numbers not individual patient data so we cannot make population‐based estimates. Further, it is not known to what extent observations reflect Australian population ageing. The proportion of people aged 60+ in Australia has increased by a factor of 1.3 between 2004 and 2021 (most recent census data) [14]. Although not directly comparable due to different preparation of data sets, the proportion of people aged 60+ accessing treatment for meth/amphetamine increased by a factor of 6.1 over the same period. While observations made in this analysis are partially explained by the ageing Australian population, it is unlikely that the increase is entirely determined by the population ageing.

Study findings align with existing evidence on meth/amphetamine‐related mortality and injecting drug use, which show higher rates of substance use and mortality among older Australians [15, 16], and the results of this study indicate that these trends are also reflected in treatment utilisation. People with MAUD delay accessing care for personal and structural reasons including stigma [17], potentially accounting for observed increases in older people accessing care. While this may partially explain observations, age of meth/amphetamine initiation has also been steadily increasing over the past 20 years [15]. The continued ageing of this population may be considered a success in that people who use meth/amphetamine are surviving to older age and still accessing care.

It is important to note however that treatment services are not currently designed to address stimulant use disorder among older adults including changing social contexts, cognition, and longer duration of use. While guidelines for treating substance use in older adults in general are available [18], said guidelines are over 15 years old and there are no specific guidelines addressing stimulant use. Australian stimulant treatment guidelines acknowledge cardiovascular and cerebrovascular risks [19], however, they provide little guidance on managing stimulant use in older adults or on age‐related changes in risk. This may delay essential cardiovascular screening among people who use meth/amphetamine, highlighting the need for guidelines to recommend earlier screening in this population.

Treatment guidelines addressing common coexisting conditions that are increasingly prevalent with age, such as hypertension, do not address illicit substance use. The *2023 Australian guideline for assessing and managing cardiovascular disease risk* provides updated recommendations, including lifestyle interventions such as tobacco smoking cessation and reduced alcohol use [20]. Despite strong links between meth/amphetamine use and cardiovascular disease and the high prevalence of MAUD in Australia [6, 21], the guideline does not address amphetamine‐type stimulant use. Formal recommendations to screen for stimulant use and inclusion of stimulant use in risk matrices are therefore warranted.

The disconnect between stimulant and cardiovascular treatment guidelines is critical: stimulant use increases cardiovascular risk, and people with meth/amphetamine‐induced cardiomyopathy are younger yet experience higher hospitalisation, morbidity and mortality rates than those with other cardiomyopathies [22]. Future updates to both guideline areas should address these combined risks in the context of Australia's ageing population of people who use meth/amphetamine.

Greater support for involvement of primary care in managing co‐occurring conditions as people age may help address this gap. In Australia, primary care coordinates complex chronic disease management and provides holistic, integrated care across specialities [23], positioning it well to support substance use management in older adults. Treatment guidelines across addiction, cardiovascular, cerebrovascular and geriatric care should therefore explicitly recommend involving primary care in managing substance use as people age.

There is scope for greater integration of geriatric and addiction medicine through a framework combining geriatric and harm reduction principles. Such a framework would support screening and treatment of substance use disorders in geriatric care while integrating geriatric expertise into addiction medicine practices through management of co‐morbidities and the incorporation of harm reduction principles of acceptance, non‐judgmental care, and that substance use need not be ceased for care to be accessed. Implementing this across the healthcare system and the life span may go some way to addressing age‐related complications of non‐medical stimulant use [24]. Importantly, stigma reduction for both substance use and ageing is required to support meaningful engagement and assessment.

## 5 Conclusions

The number and proportion of older Australians accessing treatment for meth/amphetamine use are at their highest levels in two decades and are likely to continue rising. However, treatment services and clinical guidelines are not yet equipped to meet the needs of older adults, underscoring the need for service adaptation to reduce meth/amphetamine‐related harms in this population.

## Author Contributions

Each author certifies that their contribution to this work meets the standards of the International Committee of Medical Journal Editors.

## Funding

The authors have nothing to report.

## Conflicts of Interest

The authors declare no conflicts of interest.

## Data Availability

This study used publicly available data from The Alcohol and Other Drug Treatment Services National Minimum Data Set (AODTS NMDS), hosted by the Australian Institute of Health and Welfare. Data may be accessed at https://dataexplorer.aihw.gov.au.
